# Hydrogen-bonding chain and dimer motifs in pyridinium and morpholinium hydrogen oxalate salts

**DOI:** 10.1107/S2056989018015827

**Published:** 2018-11-16

**Authors:** David Z. T. Mulrooney, Eimear C. Madden, Rhona F. Lonergan, Valentyna D. Slyusarchuk, Helge Müller-Bunz, Tony D. Keene

**Affiliations:** a School of Chemistry, University College Dublin, Belfield, Dublin 4, Ireland

**Keywords:** oxalate, hydrogen bonding, ammonium cations, crystal structure

## Abstract

We present here three compounds consisting of pyridinium or morpholinium hydrogen oxalates, each displaying different hydrogen-bonding motifs, resulting in chains for 4-(di­methyl­amino)­pyridinium hydrogen oxalate 0.22-hydrate, C_7_H_11_N_2_^+^·C_2_HO_4_^−^·0.22H_2_O (**1**), dimers for 4-*tert*-butyl­pyridinium hydrogen oxalate, C_9_H_14_N^+^·C_2_HO_4_^−^ (**2**), and chains for morpholin­ium hydrogen oxalate, C_4_H_10_NO^+^·C_2_HO_4_^−^ (**3**).

## Abstract 2

Three compounds consisting of pyridinium or morpholinium hydrogen oxalates each display different hydrogen oxalate hydrogen-bonding motifs, resulting in chains for 4-(di­methyl­amino)­pyridinium hydrogen oxalate 0.22-hydrate, dimers for 4-*tert*-butyl­pyridinium hydrogen oxalate and chains for morpholinium hydrogen oxalate.

## Chemical context   

Oxalate is a common ligand in coordination chemistry, utilized for its ability to chelate and bridge metal ions to form complexes and coordination polymers (Decurtins, 1999 ▸). Its ability to facilitate strong magnetic inter­actions and stability under differing synthetic conditions makes it a ligand of choice for the rational design of magnetic materials (Pilkington & Decurtins, 2003 ▸). As the simplest di­carb­oxy­lic acid, it can also be found in differing states of deprotonation, providing a range of hydrogen-bonding motifs. Oxalate also has the unusual property of containing a C—C bond with a bond order of slightly less than one, resulting in the carboxyl­ate moieties taking a perpendicular orientation in gas phase calculations (Herbert & Ortiz, 2000 ▸). While this structure is the most energetically favourable, the difference in energy between the 90° and 0° torsion angles is slight and is often overridden in hydrogen-bonded structures. Ammonium hydrogen oxalate salts are often useful precursors in the formation of transition metal complexes (Keene *et al.*, 2003 ▸) and coordination polymers (Keene *et al.*, 2004 ▸). Our research group has an inter­est in these precursors as part of our investigations into mol­ecular magnets (Keene, *et al.* 2010 ▸), not only for their usefulness in this role, but for the complex hydrogen-bonded structures that often arise on crystallization. Previous work from our group has focused on the structure of discrete oxalate dianions and drawn correlations between torsion angles, bond lengths and the crystal packing (Keene *et al.*, 2012 ▸).

## Structural commentary   

**Figure 1:**
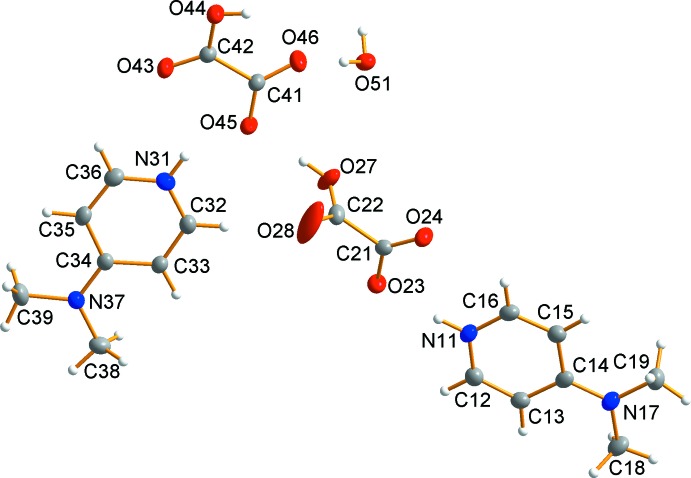
Asymmetric unit of **1**. Displacement ellipsoids are drawn at the 50% probability level.

Compound **1** crystallizes in the triclinic space group *P* 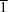. The asymmetric unit of **1** (Fig. 1 ▸) consists of two 4-di­methyl­amino­pyridinium cations, two hydrogen oxalate anions and a partial-occupancy water mol­ecule [44.3 (4)% occupancy]. The two hydrogen oxalate anions show markedly different structures with the C21–C22 moiety displaying almost perpendic­ular O—C—C—O torsion angles of −82.784 (9) and −81.855 (10)° while C41—C42 is closer to planar with torsion angles of −13.267 (11) and −12.915 (10)°. The C—C bonds (Table 1 ▸) are consistent with other oxalate anions being 1.5276 (18) Å for C21–C22 and 1.5527 (18) Å for C41–C42.
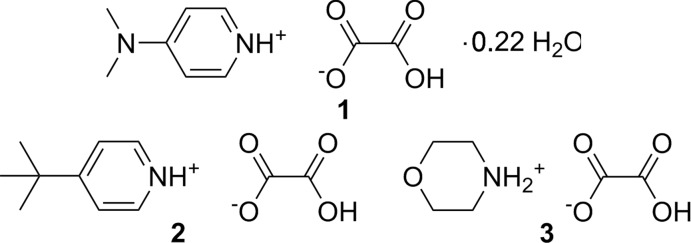


**Figure 2:**
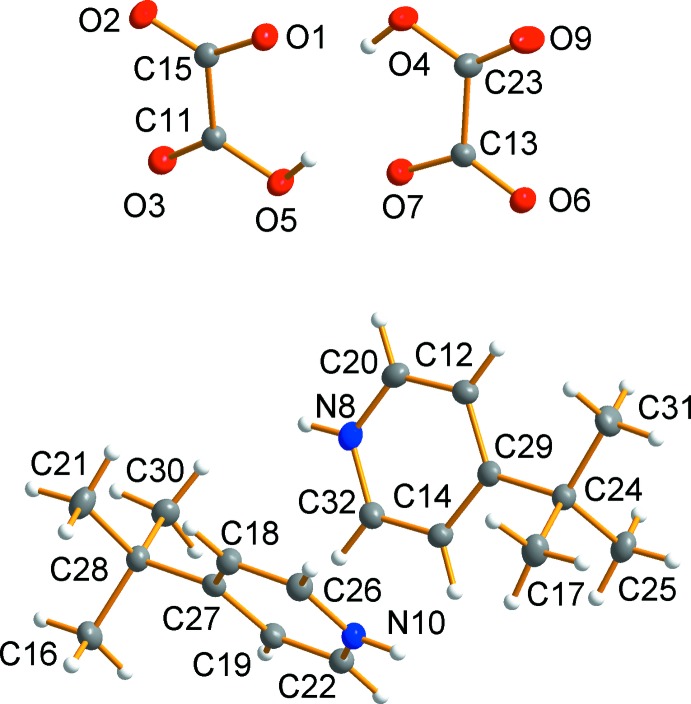
Asymmetric unit of **2**. Displacement ellipsoids are drawn at the 50% probability level.

Compound **2** crystallizes in the monoclinic space group *P*2_1_/*c*. The asymmetric unit of **2** (Fig. 2 ▸) consists of two 4-*t*-butyl­pyridinium cations and two hydrogen oxalate anions. Both of the hydrogen oxalate moieties are nearly planar with torsion angles of 1.39 (13)° and 1.58 (15)° for C11—C15 and 1.93 (14)° and 2.73 (15)° for C13—C23.

**Figure 3:**
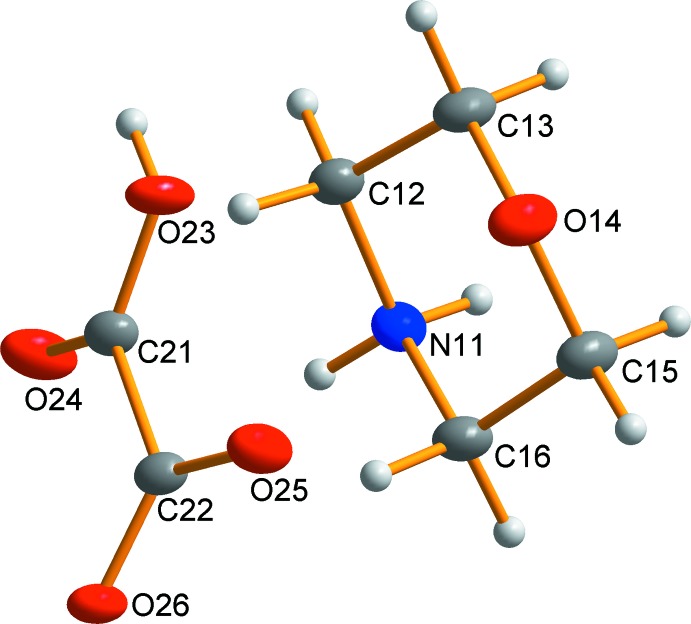
Asymmetric unit of **3**. Displacement ellipsoids are drawn at the 50% probability level.

Compound **3** crystallizes in the monoclinic space group *P*2_1_/*c*. The asymmetric unit of **3** (Fig. 3 ▸) consists of one morpholinium cation and one hydrogen oxalate anion. The hydrogen oxalate moiety is near to planar with torsion angles of −11.3 (2) and −12.0 (2)°.

## Supra­molecular features   

**Figure 4:**
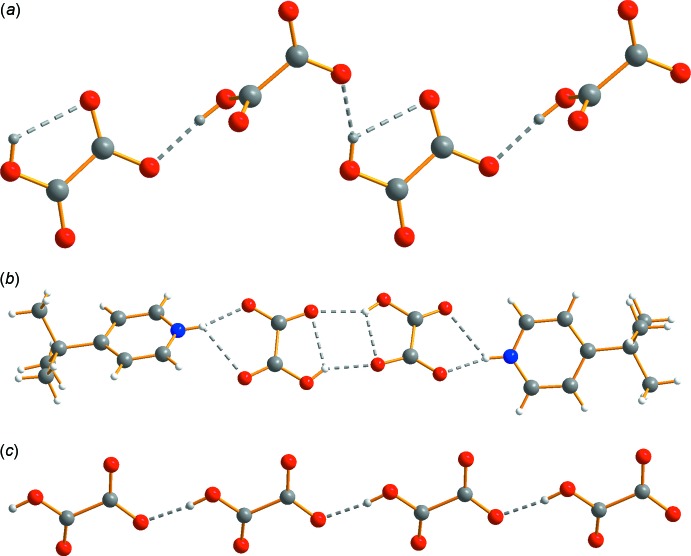
Hydrogen bonding in hydrogen oxalate groups: (*a*) chain formed in compound **1**, (*b*) hydrogen-bonded dimer tecton in compound **2** and (*c*) chain formed in compound **3**. **[Please include the cell axes]**

Each of the salts displays a hydrogen-bonded network, building the three-dimensional structure of the crystal (Fig. 4 ▸). In compound **1**, every oxygen atom of the hydrogen oxalates and water groups takes part in hydrogen bonds (Table 2 ▸). Extensive C—H⋯O inter­actions and π–π stacking [*Cg*1⋯*Cg*1(2 − *x*, −*y*, 2 − *z*) = 3.6418 (8) Å and *Cg2*⋯*Cg*2(2 − *x*, 1 − *y*, 1 − *z*) = 3.6535 (9) Å; *Cg*1 and *Cg*2 are the centroids of the N11/C12–C16 and N31/C32–C36 rings, respectively] complete the inter­molecular inter­actions. The hydrogen oxalate moieties form a hydrogen-bonded chain along the [1
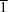
0] direction.

In compound **2**, the hydrogen oxalate moieties form hydrogen-bonded pairs (Table 3 ▸) with a four-membered ring formed at the centre of the pair. The opposite sides of the oxalates form a bifurcated hydrogen bond to the 4-*t*-butyl­pyridinium groups, generating a supra­molecular tecton. These are then built into the three-dimensional structure through C—H⋯O inter­actions. The presence of the *t*-butyl groups suppresses π–π stacking due to steric inter­ference with no obvious C—H⋯π inter­actions present.

In compound **3**, the hydrogen oxalates form a chain along the *a*-axis direction. These chains form the core of the structure with hydrogen bonds (Table 4 ▸) coming from the morpholinium along with C—H⋯O inter­actions that form the three-dimensional structure.

## Database survey   

Hydrogen-bonding motifs in hydrogen oxalate compounds often tend towards chain formation. Different chain types are formed depending on the conformation of the hydroxyl group, *i.e*. whether the O—H bond is *cis* or *trans* to the C—C bond. In compound **3**, the hydrogen oxalate is the *trans* conformer and produces a chain along the *a*-axis direction and is comparable to compounds reported in the Cambridge Structural Database (CSD version 5.39, updated August 2018, Groom *et al.*, 2016 ▸), such as ACOQER (Mora *et al.*, 2017 ▸) and FOMBIU (Traut-Johnstone *et al.*, 2014 ▸). The hydrogen oxalates in compound **2** are in the *cis* conformation and form a hydrogen-bonded pair, as seen in a small handful of structures: the combination of this pair-wise inter­action with a birfurcated hydrogen bond to a pyridinium cation is also seen in EZECOC (Androš *et al.*, 2011 ▸; Chen *et al.* 2012 ▸,), GULQOV (Thomas *et al.*, 2015 ▸; Suresh *et al.*, 2015 ▸), LOFMAW (Hu *et al.*, 2014 ▸), YEPBAX (Said *et al.*, 2006 ▸), YINVUO (Martin *et al.*, 2013 ▸) and XEJRIQ (Edwards & Schafer, 2017 ▸). The chain type in **1** is not seen in any hydrogen oxalate compounds in the CSD.

## Synthesis and crystallization   

Compound **1** was synthesized by adding a solution of 4-di­methyl­amino­pyridine (1.0 mmol, 122 mg) in water (10 ml) and oxalic acid dihydrate (126 mg, 1.0 mmol) in water (10 ml). The resultant solution was left to evaporate to a white powder and was then recrystallized from hot aceto­nitrile to give colourless crystals suitable for single-crystal X-ray diffraction.

The synthesis of compound **2** was achieved by addition of anhydrous oxalic acid (900 mg, 10 mmol) in distilled water (10 ml) to a non-miscible mixture of 4-*t*-butyl­pyridine (1.465ml, 10 mmol) and distilled water (10 ml) to give a homogenous solution. This was left to evaporate over five days and the white product recrystallized from hot methanol.

Compound **3** was synthesized by adding a solution of oxalic acid dihydrate (1271 mg, 10 mmol) in water (10 ml) to a solution of morpholine (862 µl, 871 mg, 10 mmol) in water (10 ml) and leaving the resultant solution to evaporate until crystals had formed.

## Refinement   

Crystal data, data collection and structure refinement details are summarized in Table 5 ▸. In all cases, the proton of the hydrogen oxalate was placed according to C—O bond lengths (O—H = 0.84 Å). All other H atoms were positioned geometrically (N—H = 0.88, O—H = 0.97, C—H = 0.95–0.98 Å) and refined as riding with *U*_iso_(H) = *kU*_eq_(parent atom) where *k* = 1.2 for all C—H and N—H groups and 1.5 for Cmethyl, Ohy­droxy and Owater.

The occupancy of the water mol­ecule in compound **1** was allowed to refine freely to 0.443 (4). Attempts to split the O27/O28 carboxyl­ate in **1** were unsuccessful, leading to a poor-quality refinement. Attempts to locate extra symmetry in compound **2** were unsuccessful, despite superficially appearing to have an inversion centre between the 4-tbpy moieties and between the hydrogen oxalate moieties.

## Supplementary Material

Crystal structure: contains datablock(s) 1, 2, 3. DOI: 10.1107/S2056989018015827/qm2130sup1.cif

Structure factors: contains datablock(s) 1. DOI: 10.1107/S2056989018015827/qm21301sup2.hkl

Structure factors: contains datablock(s) 2. DOI: 10.1107/S2056989018015827/qm21302sup3.hkl

Structure factors: contains datablock(s) 3. DOI: 10.1107/S2056989018015827/qm21303sup4.hkl

CCDC references: 1877733, 1877732, 1877731

Additional supporting information:  crystallographic information; 3D view; checkCIF report

## Figures and Tables

**Table 1:** Selected geometric parameters (Å, °) for (1)

C21—O23	1.2639 (16)	C22—O28	1.196 (2)
C21—O24	1.2310 (17)	C22—O27	1.2976 (19)
C21—C22	1.5276 (18)	C41—C42	1.5527 (18)
			
O24—C21—O23	126.89 (13)	O46—C41—O45	127.39 (13)
O28—C22—O27	125.39 (14)	O43—C42—O44	122.19 (12)

**Table 2:** Hydrogen-bond geometry (Å, °) for **1** [Chem scheme1]

*D*—H⋯*A*	*D*—H	H⋯*A*	*D*⋯*A*	*D*—H⋯*A*
O27—H27⋯O45	0.84	1.72	2.553 (2)	171
O44—H44⋯O23^i^	0.84	1.84	2.645 (2)	160
N31—H31⋯O45	0.88	1.87	2.672 (2)	151
N11—H11⋯O23	0.88	1.87	2.749 (2)	174

Symmetry code: (i) 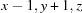.

**Table 3:** Hydrogen-bond geometry (Å, °) for **2** [Chem scheme1]

*D*—H⋯*A*	*D*—H	H⋯*A*	*D*⋯*A*	*D*—H⋯*A*
O5—H5⋯O1	0.84	2.22	2.702 (2)	116
O5—H5⋯O7	0.84	1.89	2.621 (2)	144
O4—H4⋯O1	0.84	1.95	2.667 (2)	143
O4—H4⋯O7	0.84	2.17	2.665 (2)	117
N8—H8⋯O6^i^	0.88	1.80	2.635 (2)	159
N10—H10⋯O2^ii^	0.88	1.84	2.691 (2)	162

Symmetry codes: (i) 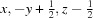; (ii) 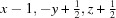.

**Table 4:** Hydrogen-bond geometry (Å, °) for **3** [Chem scheme1]

*D*—H⋯*A*	*D*—H	H⋯*A*	*D*⋯*A*	*D*—H⋯*A*
O23—H23⋯O26^i^	0.84	1.75	2.587 (2)	173
N11—H11*A*⋯O26^ii^	0.91	2.06	2.879 (2)	149
N11—H11*B*⋯O25^iii^	0.91	1.92	2.773 (2)	156

Symmetry codes: (i) 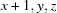; (ii) 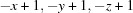; (iii) 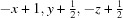.

**Table 5:** Experimental details

	**1**	**2**	**3**
Crystal data			
Chemical formula	C_7_H_11_N_2_^+^·C_2_HO_4_^−^·0.22H_2_O	C_9_H_14_N^+^·C_2_HO_4_^−^	C_4_H_10_NO^+^·C_2_HO_4_^−^
*M*_r_	216.21	225.24	177.16
Crystal system, space group	Triclinic, *P* 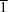	Monoclinic, *P*2_1_/*c*	Monoclinic, *P*2_1_/*c*
Temperature (K)	101	101	100
*a*, *b*, *c* (Å)	7.5241 (3), 8.2898 (3), 18.7359 (6)	9.7043 (1), 20.6128 (2), 11.3649 (2)	5.6867 (3), 12.2465 (8), 12.0831 (6)
α, β, γ (°)	89.738 (3), 79.626 (3), 64.741 (4)	90, 95.301 (1), 90	90, 113.150 (4), 90
*V* (Å^3^)	1036.17 (7)	2263.63 (5)	773.73 (8)
*Z*	4	8	4
Radiation type	Cu *K*α	Cu *K*α	Mo *K*α
μ (mm^−1^)	0.95	0.84	0.13
Crystal size (mm)	0.22 × 0.12 × 0.12	0.23 × 0.21 × 0.15	0.12 × 0.08 × 0.06

Data collection			
Diffractometer	Rigaku SuperNova, Dual, Cu at zero, Atlas	Rigaku SuperNova, Dual, Cu at zero, Atlas	Nonius Kappa CCD
Absorption correction	Gaussian (*CrysAlis PRO*; Rigaku OD, 2017 ▸)	Gaussian (*CrysAlis PRO*; Rigaku OD, 2017 ▸)	Multi-scan (*SORTAV*; Blessing, 1997 ▸)
*T*_min_, *T*_max_	0.857, 0.918	0.875, 0.914	0.887, 1.175
No. of measured, independent and observed [*I* > 2σ(*I*)] reflections	12774, 4321, 3792	23245, 4749, 4309	6288, 1769, 1390
*R*_int_	0.024	0.026	0.075
(sin θ/λ)_max_ (Å^−1^)	0.631	0.632	0.652

Refinement			
*R*[*F* ^2^ > 2σ(*F* ^2^)], *wR*(*F* ^2^), *S*	0.039, 0.102, 1.03	0.032, 0.086, 1.03	0.042, 0.110, 1.05
No. of reflections	4321	4749	1769
No. of parameters	290	298	111
H-atom treatment	H-atom parameters constrained	H-atom parameters constrained	H-atom parameters constrained
Δρ_max_, Δρ_min_ (e Å^−3^)	0.64, −0.61	0.30, −0.20	0.29, −0.27

Computer programs: *CrysAlis PRO* (Rigaku OD, 2017 ▸), *DENZO* (Otwinowski & Minor, 1997 ▸), *COLLECT* (Hooft, 1998 ▸), *SHELXT* (Sheldrick, 2015*a* ▸), *SHELXS97* (Sheldrick, 2008 ▸), *SHELXL2018* (Sheldrick, 2015*b* ▸) and *OLEX2* (Dolomanov *et al.*, 2009 ▸).

## supplementary crystallographic information

### 4-(Dimethylamino)pyridinum hydrogen oxalate 0.22-hydrate, (1) Crystal data

**Table:**

C_7_H_11_N_2_^+^·C_2_HO_4_^−^·0.22H_2_O	*Z* = 4
*M**_r_* = 216.21	*F*(000) = 457
Triclinic, *P*1	*D*_x_ = 1.386 Mg m^−^^3^
*a* = 7.5241 (3) Å	Cu *K*α radiation, λ = 1.54184 Å
*b* = 8.2898 (3) Å	Cell parameters from 6456 reflections
*c* = 18.7359 (6) Å	θ = 4.8–76.7°
α = 89.738 (3)°	µ = 0.95 mm^−^^1^
β = 79.626 (3)°	*T* = 101 K
γ = 64.741 (4)°	Block, colourless
*V* = 1036.17 (7) Å^3^	0.22 × 0.12 × 0.12 mm

### 4-(Dimethylamino)pyridinum hydrogen oxalate 0.22-hydrate, (1) Data collection

**Table:**

Rigaku SuperNova, Dual, Cu at zero, Atlas diffractometer	4321 independent reflections
Radiation source: micro-focus sealed X-ray tube, SuperNova (Cu) X-ray Source	3792 reflections with *I* > 2σ(*I*)
Mirror monochromator	*R*_int_ = 0.024
Detector resolution: 10.3196 pixels mm^-1^	θ_max_ = 76.8°, θ_min_ = 4.8°
ω scans	*h* = −9→9
Absorption correction: gaussian (CrysAlis PRO; Rigaku OD, 2017)	*k* = −10→10
*T*_min_ = 0.857, *T*_max_ = 0.918	*l* = −23→23
12774 measured reflections	

### 4-(Dimethylamino)pyridinum hydrogen oxalate 0.22-hydrate, (1) Refinement

**Table:**

Refinement on *F*^2^	Hydrogen site location: mixed
Least-squares matrix: full	H-atom parameters constrained
*R*[*F*^2^ > 2σ(*F*^2^)] = 0.039	*w* = 1/[σ^2^(*F*_o_^2^) + (0.0459*P*)^2^ + 0.4606*P*] where *P* = (*F*_o_^2^ + 2*F*_c_^2^)/3
*wR*(*F*^2^) = 0.102	(Δ/σ)_max_ = 0.001
*S* = 1.03	Δρ_max_ = 0.64 e Å^−^^3^
4321 reflections	Δρ_min_ = −0.60 e Å^−^^3^
290 parameters	Extinction correction: SHELXL2017 (Sheldrick, 2015b), Fc^*^=kFc[1+0.001xFc^2^λ^3^/sin(2θ)]^-1/4^
0 restraints	Extinction coefficient: 0.0050 (6)
Primary atom site location: dual	

### 4-(Dimethylamino)pyridinum hydrogen oxalate 0.22-hydrate, (1) Special details

**Table:**

Geometry. All esds (except the esd in the dihedral angle between two l.s. planes) are estimated using the full covariance matrix. The cell esds are taken into account individually in the estimation of esds in distances, angles and torsion angles; correlations between esds in cell parameters are only used when they are defined by crystal symmetry. An approximate (isotropic) treatment of cell esds is used for estimating esds involving l.s. planes.

### 4-(Dimethylamino)pyridinum hydrogen oxalate 0.22-hydrate, (1) Fractional atomic coordinates and isotropic or equivalent isotropic displacement parameters (Å^2^)

**Table:**

	*x*	*y*	*z*	*U*_iso_*/*U*_eq_	Occ. (<1)
C21	0.63947 (19)	0.24757 (17)	0.81649 (7)	0.0209 (3)	
O23	0.81556 (15)	0.12920 (14)	0.79288 (5)	0.0291 (2)	
O24	0.53451 (15)	0.26088 (13)	0.87673 (5)	0.0291 (2)	
C22	0.5487 (2)	0.38424 (19)	0.76292 (7)	0.0252 (3)	
O28	0.4407 (3)	0.3678 (3)	0.72680 (10)	0.0782 (6)	
O27	0.6027 (2)	0.51364 (15)	0.76121 (7)	0.0423 (3)	
H27	0.543889	0.587961	0.732904	0.063*	
C41	0.31016 (19)	0.88186 (18)	0.69597 (7)	0.0221 (3)	
C42	0.2033 (2)	1.00318 (18)	0.63889 (7)	0.0229 (3)	
O43	0.28018 (16)	0.97891 (14)	0.57487 (5)	0.0304 (2)	
O44	0.02609 (15)	1.13493 (14)	0.66442 (5)	0.0287 (2)	
H44	−0.013467	1.120732	0.707824	0.043*	
O45	0.46062 (17)	0.73901 (14)	0.66943 (6)	0.0362 (3)	
O46	0.24268 (16)	0.93378 (15)	0.76030 (5)	0.0334 (3)	
N31	0.64826 (18)	0.59063 (16)	0.53471 (7)	0.0265 (3)	
H31	0.559425	0.667206	0.570245	0.032*	
C32	0.7864 (2)	0.43279 (19)	0.55026 (7)	0.0255 (3)	
H32	0.785917	0.406403	0.599668	0.031*	
C33	0.9265 (2)	0.31050 (18)	0.49705 (7)	0.0230 (3)	
H33	1.022104	0.200326	0.509378	0.028*	
C34	0.9289 (2)	0.34848 (17)	0.42281 (7)	0.0218 (3)	
C35	0.7797 (2)	0.51610 (18)	0.40884 (7)	0.0247 (3)	
H35	0.773915	0.547156	0.360160	0.030*	
C36	0.6455 (2)	0.63162 (19)	0.46511 (8)	0.0266 (3)	
H36	0.547814	0.743611	0.455160	0.032*	
N37	1.06573 (19)	0.23242 (16)	0.36900 (6)	0.0265 (3)	
C38	1.2241 (2)	0.0656 (2)	0.38514 (8)	0.0308 (3)	
H38A	1.306530	−0.004623	0.339489	0.046*	
H38B	1.163647	−0.003441	0.414125	0.046*	
H38C	1.308285	0.093268	0.412603	0.046*	
C39	1.0741 (3)	0.2804 (2)	0.29357 (8)	0.0346 (3)	
H39A	1.177294	0.178370	0.261209	0.052*	
H39B	1.106919	0.382807	0.288657	0.052*	
H39C	0.943665	0.312091	0.280341	0.052*	
N11	0.93551 (19)	−0.08918 (16)	0.90258 (6)	0.0271 (3)	
H11	0.905583	−0.023294	0.865684	0.033*	
C12	1.1096 (2)	−0.23848 (19)	0.89417 (8)	0.0279 (3)	
H12	1.198139	−0.271263	0.848133	0.034*	
C13	1.1617 (2)	−0.34363 (18)	0.95003 (8)	0.0253 (3)	
H13	1.284904	−0.448706	0.942505	0.030*	
C14	1.0325 (2)	−0.29652 (18)	1.01939 (7)	0.0232 (3)	
C15	0.8495 (2)	−0.13857 (18)	1.02576 (8)	0.0262 (3)	
H15	0.756700	−0.101448	1.070848	0.031*	
C16	0.8072 (2)	−0.04046 (18)	0.96725 (8)	0.0269 (3)	
H16	0.684523	0.064542	0.972233	0.032*	
N17	1.07977 (18)	−0.39505 (16)	1.07586 (7)	0.0262 (3)	
C18	1.2723 (2)	−0.5515 (2)	1.06937 (9)	0.0313 (3)	
H18A	1.380322	−0.513174	1.062420	0.047*	
H18B	1.291850	−0.631894	1.027518	0.047*	
H18C	1.273973	−0.614446	1.113826	0.047*	
C19	0.9438 (2)	−0.3446 (2)	1.14685 (8)	0.0323 (3)	
H19A	0.815307	−0.342245	1.141889	0.048*	
H19B	0.922145	−0.225643	1.165034	0.048*	
H19C	1.002919	−0.431983	1.181181	0.048*	
O51	0.5352 (3)	0.9043 (3)	0.84705 (12)	0.0266 (7)	0.443 (4)
H51A	0.525878	1.012002	0.852157	0.040*	0.443 (4)
H51B	0.451198	0.913256	0.819034	0.040*	0.443 (4)

### 4-(Dimethylamino)pyridinum hydrogen oxalate 0.22-hydrate, (1) Atomic displacement parameters (Å^2^)

**Table:**

	*U*^11^	*U*^22^	*U*^33^	*U*^12^	*U*^13^	*U*^23^
C21	0.0200 (6)	0.0189 (6)	0.0233 (6)	−0.0069 (5)	−0.0074 (5)	0.0009 (5)
O23	0.0220 (5)	0.0278 (5)	0.0269 (5)	−0.0012 (4)	−0.0041 (4)	0.0030 (4)
O24	0.0244 (5)	0.0274 (5)	0.0272 (5)	−0.0045 (4)	−0.0021 (4)	0.0080 (4)
C22	0.0193 (6)	0.0309 (7)	0.0211 (6)	−0.0061 (5)	−0.0053 (5)	0.0044 (5)
O28	0.1000 (13)	0.1124 (14)	0.0845 (12)	−0.0817 (12)	−0.0781 (11)	0.0698 (11)
O27	0.0641 (8)	0.0266 (6)	0.0474 (7)	−0.0205 (6)	−0.0360 (6)	0.0180 (5)
C41	0.0213 (6)	0.0214 (6)	0.0222 (6)	−0.0077 (5)	−0.0051 (5)	0.0044 (5)
C42	0.0231 (6)	0.0210 (6)	0.0233 (6)	−0.0077 (5)	−0.0060 (5)	0.0038 (5)
O43	0.0304 (5)	0.0302 (5)	0.0232 (5)	−0.0064 (4)	−0.0048 (4)	0.0080 (4)
O44	0.0251 (5)	0.0274 (5)	0.0246 (5)	−0.0025 (4)	−0.0059 (4)	0.0047 (4)
O45	0.0408 (6)	0.0238 (5)	0.0241 (5)	0.0037 (5)	−0.0041 (4)	0.0051 (4)
O46	0.0276 (5)	0.0364 (6)	0.0217 (5)	−0.0001 (4)	−0.0055 (4)	0.0014 (4)
N31	0.0249 (6)	0.0253 (6)	0.0263 (6)	−0.0084 (5)	−0.0040 (5)	−0.0018 (5)
C32	0.0293 (7)	0.0274 (7)	0.0220 (6)	−0.0131 (6)	−0.0081 (5)	0.0037 (5)
C33	0.0258 (6)	0.0217 (6)	0.0224 (6)	−0.0095 (5)	−0.0090 (5)	0.0046 (5)
C34	0.0254 (6)	0.0220 (6)	0.0213 (6)	−0.0121 (5)	−0.0078 (5)	0.0027 (5)
C35	0.0281 (7)	0.0261 (7)	0.0231 (6)	−0.0124 (6)	−0.0115 (5)	0.0070 (5)
C36	0.0248 (7)	0.0237 (7)	0.0319 (7)	−0.0090 (5)	−0.0112 (5)	0.0054 (5)
N37	0.0321 (6)	0.0245 (6)	0.0208 (6)	−0.0106 (5)	−0.0046 (5)	0.0014 (4)
C38	0.0301 (7)	0.0240 (7)	0.0334 (8)	−0.0077 (6)	−0.0047 (6)	−0.0020 (6)
C39	0.0454 (9)	0.0386 (8)	0.0198 (7)	−0.0191 (7)	−0.0041 (6)	0.0018 (6)
N11	0.0347 (6)	0.0228 (6)	0.0261 (6)	−0.0115 (5)	−0.0141 (5)	0.0063 (4)
C12	0.0322 (7)	0.0270 (7)	0.0255 (7)	−0.0127 (6)	−0.0083 (6)	0.0016 (5)
C13	0.0253 (6)	0.0211 (6)	0.0285 (7)	−0.0076 (5)	−0.0092 (5)	0.0008 (5)
C14	0.0278 (7)	0.0207 (6)	0.0268 (7)	−0.0132 (5)	−0.0127 (5)	0.0043 (5)
C15	0.0293 (7)	0.0230 (7)	0.0264 (7)	−0.0100 (6)	−0.0085 (5)	0.0011 (5)
C16	0.0292 (7)	0.0200 (6)	0.0315 (7)	−0.0081 (5)	−0.0125 (6)	0.0021 (5)
N17	0.0286 (6)	0.0251 (6)	0.0278 (6)	−0.0122 (5)	−0.0116 (5)	0.0075 (5)
C18	0.0317 (7)	0.0270 (7)	0.0376 (8)	−0.0109 (6)	−0.0179 (6)	0.0101 (6)
C19	0.0384 (8)	0.0353 (8)	0.0256 (7)	−0.0167 (7)	−0.0100 (6)	0.0065 (6)
O51	0.0255 (12)	0.0223 (12)	0.0306 (13)	−0.0075 (9)	−0.0093 (9)	0.0031 (9)

### 4-(Dimethylamino)pyridinum hydrogen oxalate 0.22-hydrate, (1) Geometric parameters (Å, º)

**Table:**

C21—O23	1.2639 (16)	C38—H38B	0.9800
C21—O24	1.2310 (17)	C38—H38C	0.9800
C21—C22	1.5276 (18)	C39—H39A	0.9800
C22—O28	1.196 (2)	C39—H39B	0.9800
C22—O27	1.2976 (19)	C39—H39C	0.9800
O27—H27	0.8400	N11—H11	0.8800
C41—C42	1.5527 (18)	N11—C12	1.3476 (19)
C41—O45	1.2608 (17)	N11—C16	1.3467 (19)
C41—O46	1.2222 (17)	C12—H12	0.9500
C42—O43	1.2112 (17)	C12—C13	1.363 (2)
C42—O44	1.3161 (16)	C13—H13	0.9500
O44—H44	0.8400	C13—C14	1.418 (2)
N31—H31	0.8800	C14—C15	1.4243 (19)
N31—C32	1.3500 (19)	C14—N17	1.3388 (18)
N31—C36	1.3476 (19)	C15—H15	0.9500
C32—H32	0.9500	C15—C16	1.364 (2)
C32—C33	1.360 (2)	C16—H16	0.9500
C33—H33	0.9500	N17—C18	1.4593 (18)
C33—C34	1.4234 (18)	N17—C19	1.4630 (19)
C34—C35	1.4251 (19)	C18—H18A	0.9800
C34—N37	1.3386 (18)	C18—H18B	0.9800
C35—H35	0.9500	C18—H18C	0.9800
C35—C36	1.360 (2)	C19—H19A	0.9800
C36—H36	0.9500	C19—H19B	0.9800
N37—C38	1.4641 (19)	C19—H19C	0.9800
N37—C39	1.4641 (18)	O51—H51A	0.8694
C38—H38A	0.9800	O51—H51B	0.8714
			
O23—C21—C22	115.43 (11)	N37—C39—H39A	109.5
O24—C21—O23	126.89 (13)	N37—C39—H39B	109.5
O24—C21—C22	117.66 (11)	N37—C39—H39C	109.5
O28—C22—C21	121.46 (14)	H39A—C39—H39B	109.5
O28—C22—O27	125.39 (14)	H39A—C39—H39C	109.5
O27—C22—C21	113.14 (12)	H39B—C39—H39C	109.5
C22—O27—H27	109.5	C12—N11—H11	119.9
O45—C41—C42	114.75 (11)	C16—N11—H11	119.9
O46—C41—C42	117.86 (12)	C16—N11—C12	120.22 (12)
O46—C41—O45	127.39 (13)	N11—C12—H12	119.2
O43—C42—C41	121.78 (12)	N11—C12—C13	121.67 (14)
O43—C42—O44	122.19 (12)	C13—C12—H12	119.2
O44—C42—C41	116.02 (11)	C12—C13—H13	119.9
C42—O44—H44	109.5	C12—C13—C14	120.14 (13)
C32—N31—H31	119.8	C14—C13—H13	119.9
C36—N31—H31	119.8	C13—C14—C15	116.37 (12)
C36—N31—C32	120.39 (12)	N17—C14—C13	121.90 (13)
N31—C32—H32	119.1	N17—C14—C15	121.73 (13)
N31—C32—C33	121.76 (13)	C14—C15—H15	119.9
C33—C32—H32	119.1	C16—C15—C14	120.14 (13)
C32—C33—H33	120.2	C16—C15—H15	119.9
C32—C33—C34	119.66 (13)	N11—C16—C15	121.46 (13)
C34—C33—H33	120.2	N11—C16—H16	119.3
C33—C34—C35	116.72 (12)	C15—C16—H16	119.3
N37—C34—C33	121.39 (12)	C14—N17—C18	121.00 (12)
N37—C34—C35	121.88 (12)	C14—N17—C19	121.01 (12)
C34—C35—H35	120.0	C18—N17—C19	117.93 (12)
C36—C35—C34	120.06 (13)	N17—C18—H18A	109.5
C36—C35—H35	120.0	N17—C18—H18B	109.5
N31—C36—C35	121.40 (13)	N17—C18—H18C	109.5
N31—C36—H36	119.3	H18A—C18—H18B	109.5
C35—C36—H36	119.3	H18A—C18—H18C	109.5
C34—N37—C38	120.70 (12)	H18B—C18—H18C	109.5
C34—N37—C39	120.23 (12)	N17—C19—H19A	109.5
C38—N37—C39	118.64 (12)	N17—C19—H19B	109.5
N37—C38—H38A	109.5	N17—C19—H19C	109.5
N37—C38—H38B	109.5	H19A—C19—H19B	109.5
N37—C38—H38C	109.5	H19A—C19—H19C	109.5
H38A—C38—H38B	109.5	H19B—C19—H19C	109.5
H38A—C38—H38C	109.5	H51A—O51—H51B	104.5
H38B—C38—H38C	109.5		

### 4-(Dimethylamino)pyridinum hydrogen oxalate 0.22-hydrate, (1) Hydrogen-bond geometry (Å, º)

**Table:**

*D*—H···*A*	*D*—H	H···*A*	*D*···*A*	*D*—H···*A*
O27—H27···O45	0.84	1.72	2.553 (2)	171
O44—H44···O23^i^	0.84	1.84	2.645 (2)	160
N31—H31···O45	0.88	1.87	2.672 (2)	151
C33—H33···O43^ii^	0.95	2.54	3.447 (2)	160
C35—H35···O28^iii^	0.95	2.39	3.204 (2)	143
C39—H39*A*···O51^iv^	0.98	2.54	3.367 (2)	143
N11—H11···O23	0.88	1.87	2.749 (2)	174
C12—H12···O46^ii^	0.95	2.44	3.101 (2)	126
C13—H13···O24^ii^	0.95	2.49	3.363 (2)	154
C15—H15···O51^v^	0.95	2.37	3.254 (2)	155
C16—H16···O24	0.95	2.50	3.189 (2)	129
C18—H18*C*···O51^vi^	0.98	2.41	3.204 (2)	138
C19—H19*A*···O24^vii^	0.98	2.51	3.474 (2)	167
C19—H19*B*···O23^vi^	0.98	2.66	3.355 (2)	128
C19—H19*B*···O46^v^	0.98	2.49	3.419 (2)	158

Symmetry codes: (i) *x*−1, *y*+1, *z*; (ii) *x*+1, *y*−1, *z*; (iii) −*x*+1, −*y*+1, −*z*+1; (iv) −*x*+2, −*y*+1, −*z*+1; (v) −*x*+1, −*y*+1, −*z*+2; (vi) −*x*+2, −*y*, −*z*+2; (vii) −*x*+1, −*y*, −*z*+2.

### 4-tert-Butylpyridinium hydrogen oxalate (2) Crystal data

**Table:**

C_9_H_14_N^+^·C_2_HO_4_^−^	*F*(000) = 960
*M**_r_* = 225.24	*D*_x_ = 1.322 Mg m^−^^3^
Monoclinic, *P*2_1_/*c*	Cu *K*α radiation, λ = 1.54184 Å
*a* = 9.7043 (1) Å	Cell parameters from 12822 reflections
*b* = 20.6128 (2) Å	θ = 4.3–76.7°
*c* = 11.3649 (2) Å	µ = 0.84 mm^−^^1^
β = 95.301 (1)°	*T* = 101 K
*V* = 2263.63 (5) Å^3^	Block, colourless
*Z* = 8	0.23 × 0.21 × 0.15 mm

### 4-tert-Butylpyridinium hydrogen oxalate (2) Data collection

**Table:**

Rigaku SuperNova, Dual, Cu at zero, Atlas diffractometer	4749 independent reflections
Radiation source: micro-focus sealed X-ray tube, SuperNova (Cu) X-ray Source	4309 reflections with *I* > 2σ(*I*)
Mirror monochromator	*R*_int_ = 0.026
Detector resolution: 10.3196 pixels mm^-1^	θ_max_ = 76.9°, θ_min_ = 4.3°
ω scans	*h* = −12→6
Absorption correction: gaussian (CrysAlis PRO; Rigaku OD, 2017)	*k* = −25→25
*T*_min_ = 0.875, *T*_max_ = 0.914	*l* = −14→13
23245 measured reflections	

### 4-tert-Butylpyridinium hydrogen oxalate (2) Refinement

**Table:**

Refinement on *F*^2^	Hydrogen site location: inferred from neighbouring sites
Least-squares matrix: full	H-atom parameters constrained
*R*[*F*^2^ > 2σ(*F*^2^)] = 0.032	*w* = 1/[σ^2^(*F*_o_^2^) + (0.0436*P*)^2^ + 0.6305*P*] where *P* = (*F*_o_^2^ + 2*F*_c_^2^)/3
*wR*(*F*^2^) = 0.086	(Δ/σ)_max_ = 0.001
*S* = 1.03	Δρ_max_ = 0.30 e Å^−^^3^
4749 reflections	Δρ_min_ = −0.19 e Å^−^^3^
298 parameters	Extinction correction: SHELXL2018 (Sheldrick, 2015b), Fc^*^=kFc[1+0.001xFc^2^λ^3^/sin(2θ)]^-1/4^
0 restraints	Extinction coefficient: 0.0014 (2)
Primary atom site location: structure-invariant direct methods	

### 4-tert-Butylpyridinium hydrogen oxalate (2) Special details

**Table:**

Geometry. All esds (except the esd in the dihedral angle between two l.s. planes) are estimated using the full covariance matrix. The cell esds are taken into account individually in the estimation of esds in distances, angles and torsion angles; correlations between esds in cell parameters are only used when they are defined by crystal symmetry. An approximate (isotropic) treatment of cell esds is used for estimating esds involving l.s. planes.

### 4-tert-Butylpyridinium hydrogen oxalate (2) Fractional atomic coordinates and isotropic or equivalent isotropic displacement parameters (Å^2^)

**Table:**

	*x*	*y*	*z*	*U*_iso_*/*U*_eq_	
O1	0.25550 (8)	0.43622 (3)	0.70494 (6)	0.02235 (17)	
O2	0.32304 (8)	0.44362 (4)	0.52213 (7)	0.02470 (17)	
O3	0.33459 (8)	0.31095 (4)	0.51650 (7)	0.02367 (17)	
O5	0.26234 (8)	0.30529 (3)	0.69505 (6)	0.02384 (17)	
H5	0.236580	0.331294	0.745548	0.036*	
C11	0.29745 (10)	0.33807 (5)	0.60229 (9)	0.0178 (2)	
C15	0.29057 (10)	0.41332 (5)	0.61104 (9)	0.0176 (2)	
O4	0.18857 (9)	0.46451 (4)	0.92146 (7)	0.02595 (18)	
H4	0.195934	0.439668	0.863786	0.039*	
O6	0.08701 (8)	0.32454 (3)	1.07151 (6)	0.02113 (16)	
O7	0.15176 (9)	0.33744 (4)	0.88866 (7)	0.02549 (18)	
N8	0.01187 (9)	0.13839 (4)	0.77806 (8)	0.02008 (18)	
H8	0.048459	0.141529	0.710267	0.024*	
O9	0.13464 (11)	0.45509 (4)	1.10604 (8)	0.0384 (2)	
N10	−0.58139 (9)	0.10354 (4)	0.82435 (8)	0.01965 (18)	
H10	−0.618785	0.096784	0.890936	0.024*	
C12	−0.02998 (10)	0.18412 (5)	0.96208 (9)	0.0197 (2)	
H12	−0.017786	0.219014	1.016658	0.024*	
C13	0.12757 (10)	0.35674 (5)	0.98830 (9)	0.0180 (2)	
C14	−0.11752 (10)	0.07878 (5)	0.90662 (9)	0.0190 (2)	
H14	−0.167076	0.040537	0.922749	0.023*	
C16	−0.43731 (11)	0.07692 (5)	0.41698 (9)	0.0235 (2)	
H16A	−0.537903	0.075552	0.398349	0.035*	
H16B	−0.406152	0.036516	0.456362	0.035*	
H16C	−0.392251	0.081887	0.343806	0.035*	
C17	−0.33134 (11)	0.11400 (6)	1.07311 (10)	0.0242 (2)	
H17A	−0.348024	0.073140	1.030059	0.036*	
H17B	−0.367429	0.150194	1.023517	0.036*	
H17C	−0.378245	0.112917	1.145842	0.036*	
C18	−0.56068 (10)	0.16819 (5)	0.65500 (9)	0.0195 (2)	
H18	−0.587545	0.205627	0.609813	0.023*	
C19	−0.43104 (10)	0.06991 (5)	0.68462 (9)	0.0190 (2)	
H19	−0.365858	0.039443	0.660399	0.023*	
C20	0.02689 (11)	0.18748 (5)	0.85519 (9)	0.0206 (2)	
H20	0.077167	0.225023	0.836279	0.025*	
C21	−0.44818 (12)	0.19745 (5)	0.43687 (10)	0.0252 (2)	
H21A	−0.406218	0.201309	0.361993	0.038*	
H21B	−0.420537	0.234575	0.487513	0.038*	
H21C	−0.549192	0.196699	0.421293	0.038*	
C22	−0.49071 (11)	0.06006 (5)	0.78815 (9)	0.0201 (2)	
H22	−0.467953	0.022485	0.834317	0.024*	
C23	0.14999 (11)	0.43045 (5)	1.01214 (9)	0.0225 (2)	
C24	−0.17490 (11)	0.12303 (5)	1.10438 (9)	0.0188 (2)	
C25	−0.11670 (11)	0.06294 (5)	1.17200 (9)	0.0236 (2)	
H25A	−0.158292	0.059412	1.247100	0.035*	
H25B	−0.016064	0.067113	1.187537	0.035*	
H25C	−0.138637	0.023997	1.124460	0.035*	
C26	−0.61575 (10)	0.15684 (5)	0.76097 (9)	0.0206 (2)	
H26	−0.678645	0.187189	0.789160	0.025*	
C27	−0.46546 (10)	0.12460 (5)	0.61422 (9)	0.0169 (2)	
C28	−0.39912 (10)	0.13457 (5)	0.49908 (9)	0.0182 (2)	
C29	−0.10559 (10)	0.12926 (5)	0.98989 (9)	0.0171 (2)	
C30	−0.24103 (11)	0.13659 (5)	0.52676 (10)	0.0226 (2)	
H30A	−0.208251	0.094310	0.557250	0.034*	
H30B	−0.216597	0.170110	0.586209	0.034*	
H30C	−0.197493	0.146638	0.454478	0.034*	
C31	−0.14953 (13)	0.18296 (5)	1.18272 (10)	0.0266 (2)	
H31A	−0.186910	0.221388	1.140180	0.040*	
H31B	−0.049865	0.188524	1.202940	0.040*	
H31C	−0.195574	0.177365	1.255250	0.040*	
C32	−0.05806 (11)	0.08431 (5)	0.80202 (9)	0.0204 (2)	
H32	−0.066310	0.049822	0.746359	0.025*	

### 4-tert-Butylpyridinium hydrogen oxalate (2) Atomic displacement parameters (Å^2^)

**Table:**

	*U*^11^	*U*^22^	*U*^33^	*U*^12^	*U*^13^	*U*^23^
O1	0.0307 (4)	0.0184 (3)	0.0189 (4)	0.0006 (3)	0.0073 (3)	−0.0010 (3)
O2	0.0365 (4)	0.0178 (4)	0.0214 (4)	0.0019 (3)	0.0111 (3)	0.0021 (3)
O3	0.0313 (4)	0.0191 (4)	0.0218 (4)	0.0018 (3)	0.0088 (3)	−0.0017 (3)
O5	0.0372 (4)	0.0159 (3)	0.0196 (4)	0.0009 (3)	0.0094 (3)	0.0001 (3)
C11	0.0185 (4)	0.0175 (5)	0.0174 (5)	0.0004 (3)	0.0024 (4)	0.0005 (4)
C15	0.0177 (4)	0.0173 (5)	0.0181 (5)	0.0008 (3)	0.0026 (4)	0.0002 (4)
O4	0.0394 (4)	0.0177 (4)	0.0226 (4)	−0.0059 (3)	0.0126 (3)	−0.0031 (3)
O6	0.0262 (4)	0.0187 (3)	0.0192 (4)	−0.0015 (3)	0.0063 (3)	0.0004 (3)
O7	0.0383 (4)	0.0204 (4)	0.0189 (4)	−0.0040 (3)	0.0090 (3)	−0.0024 (3)
N8	0.0217 (4)	0.0220 (4)	0.0169 (4)	0.0020 (3)	0.0041 (3)	0.0022 (3)
O9	0.0653 (6)	0.0252 (4)	0.0281 (4)	−0.0146 (4)	0.0230 (4)	−0.0097 (3)
N10	0.0224 (4)	0.0207 (4)	0.0162 (4)	−0.0006 (3)	0.0040 (3)	−0.0003 (3)
C12	0.0223 (5)	0.0162 (4)	0.0203 (5)	−0.0002 (4)	0.0007 (4)	−0.0006 (4)
C13	0.0181 (4)	0.0180 (5)	0.0179 (5)	−0.0008 (3)	0.0023 (4)	−0.0011 (4)
C14	0.0220 (5)	0.0161 (4)	0.0189 (5)	−0.0004 (4)	0.0024 (4)	0.0008 (4)
C16	0.0273 (5)	0.0262 (5)	0.0171 (5)	−0.0030 (4)	0.0036 (4)	−0.0035 (4)
C17	0.0213 (5)	0.0301 (5)	0.0214 (5)	0.0026 (4)	0.0038 (4)	0.0023 (4)
C18	0.0218 (5)	0.0182 (5)	0.0187 (5)	0.0019 (4)	0.0022 (4)	0.0011 (4)
C19	0.0221 (5)	0.0165 (4)	0.0184 (5)	0.0013 (4)	0.0021 (4)	−0.0013 (4)
C20	0.0209 (5)	0.0179 (5)	0.0230 (5)	−0.0009 (4)	0.0020 (4)	0.0036 (4)
C21	0.0299 (6)	0.0247 (5)	0.0219 (5)	0.0049 (4)	0.0072 (4)	0.0067 (4)
C22	0.0251 (5)	0.0168 (5)	0.0183 (5)	−0.0003 (4)	0.0017 (4)	0.0004 (4)
C23	0.0279 (5)	0.0196 (5)	0.0213 (5)	−0.0048 (4)	0.0086 (4)	−0.0028 (4)
C24	0.0217 (5)	0.0183 (5)	0.0165 (5)	0.0009 (4)	0.0029 (4)	0.0006 (4)
C25	0.0266 (5)	0.0244 (5)	0.0199 (5)	0.0030 (4)	0.0026 (4)	0.0060 (4)
C26	0.0217 (5)	0.0205 (5)	0.0200 (5)	0.0026 (4)	0.0033 (4)	−0.0006 (4)
C27	0.0177 (4)	0.0167 (4)	0.0162 (4)	−0.0018 (3)	0.0008 (4)	−0.0016 (3)
C28	0.0207 (5)	0.0184 (5)	0.0158 (4)	0.0008 (4)	0.0031 (4)	0.0005 (4)
C29	0.0175 (4)	0.0169 (4)	0.0166 (5)	0.0024 (3)	0.0003 (4)	0.0015 (4)
C30	0.0211 (5)	0.0243 (5)	0.0227 (5)	−0.0023 (4)	0.0036 (4)	0.0013 (4)
C31	0.0361 (6)	0.0250 (5)	0.0193 (5)	0.0003 (4)	0.0057 (4)	−0.0043 (4)
C32	0.0240 (5)	0.0183 (5)	0.0189 (5)	0.0016 (4)	0.0016 (4)	−0.0011 (4)

### 4-tert-Butylpyridinium hydrogen oxalate (2) Geometric parameters (Å, º)

**Table:**

O1—C15	1.2429 (12)	C13—C23	1.5551 (14)
O2—C15	1.2526 (12)	C14—C29	1.4041 (14)
O3—C11	1.2075 (12)	C14—C32	1.3732 (14)
O5—C11	1.3230 (12)	C16—C28	1.5350 (14)
C11—C15	1.5560 (13)	C17—C24	1.5382 (14)
O4—C23	1.3291 (13)	C18—C26	1.3820 (14)
O6—C13	1.2485 (12)	C18—C27	1.3982 (14)
O7—C13	1.2429 (12)	C19—C22	1.3735 (14)
N8—C20	1.3379 (14)	C19—C27	1.4046 (14)
N8—C32	1.3460 (14)	C21—C28	1.5310 (14)
O9—C23	1.2035 (13)	C24—C25	1.5374 (14)
N10—C22	1.3469 (13)	C24—C29	1.5243 (13)
N10—C26	1.3390 (14)	C24—C31	1.5293 (14)
C12—C20	1.3816 (15)	C27—C28	1.5248 (13)
C12—C29	1.3999 (14)	C28—C30	1.5379 (14)
			
O3—C11—O5	121.67 (9)	C25—C24—C17	109.09 (8)
O3—C11—C15	122.06 (9)	C29—C24—C17	108.48 (8)
O5—C11—C15	116.26 (8)	C29—C24—C25	108.90 (8)
O1—C15—O2	127.77 (9)	C29—C24—C31	111.65 (8)
O1—C15—C11	116.76 (9)	C31—C24—C17	109.55 (9)
O2—C15—C11	115.47 (8)	C31—C24—C25	109.13 (9)
C20—N8—C32	121.35 (9)	N10—C26—C18	120.70 (9)
C26—N10—C22	121.26 (9)	C18—C27—C19	117.17 (9)
C20—C12—C29	119.95 (9)	C18—C27—C28	122.83 (9)
O6—C13—C23	115.87 (9)	C19—C27—C28	120.00 (9)
O7—C13—O6	128.26 (9)	C16—C28—C30	109.00 (8)
O7—C13—C23	115.88 (9)	C21—C28—C16	109.11 (8)
C32—C14—C29	120.45 (9)	C21—C28—C30	109.47 (8)
C26—C18—C27	120.05 (9)	C27—C28—C16	108.66 (8)
C22—C19—C27	120.64 (9)	C27—C28—C21	111.87 (8)
N8—C20—C12	120.67 (9)	C27—C28—C30	108.68 (8)
N10—C22—C19	120.16 (9)	C12—C29—C14	117.31 (9)
O4—C23—C13	115.21 (9)	C12—C29—C24	122.86 (9)
O9—C23—O4	122.11 (10)	C14—C29—C24	119.83 (9)
O9—C23—C13	122.68 (10)	N8—C32—C14	120.26 (9)

### 4-tert-Butylpyridinium hydrogen oxalate (2) Hydrogen-bond geometry (Å, º)

**Table:**

*D*—H···*A*	*D*—H	H···*A*	*D*···*A*	*D*—H···*A*
O5—H5···O1	0.84	2.22	2.702 (2)	116
O5—H5···O7	0.84	1.89	2.621 (2)	144
O4—H4···O1	0.84	1.95	2.667 (2)	143
O4—H4···O7	0.84	2.17	2.665 (2)	117
N8—H8···O6^i^	0.88	1.80	2.635 (2)	159
N10—H10···O2^ii^	0.88	1.84	2.691 (2)	162
C12—H12···O6	0.95	2.46	3.310 (2)	149
C14—H14···O2^iii^	0.95	2.62	3.563 (2)	174
C18—H18···O3^iv^	0.95	2.50	3.446 (2)	172
C19—H19···O4^iii^	0.95	2.55	3.498 (2)	175
C20—H20···O7	0.95	2.48	3.329 (2)	148
C22—H22···O2^iii^	0.95	2.62	3.523 (2)	159
C26—H26···O3^ii^	0.95	2.58	3.060 (2)	112
C32—H32···O9^i^	0.95	2.63	3.144 (2)	114

Symmetry codes: (i) *x*, −*y*+1/2, *z*−1/2; (ii) *x*−1, −*y*+1/2, *z*+1/2; (iii) −*x*, *y*−1/2, −*z*+3/2; (iv) *x*−1, *y*, *z*.

### Morpholinium hydrogen oxalate (3) Crystal data

**Table:**

C_4_H_10_NO^+^·C_2_HO_4_^−^	*F*(000) = 376
*M**_r_* = 177.16	*D*_x_ = 1.521 Mg m^−^^3^
Monoclinic, *P*2_1_/*c*	Mo *K*α radiation, λ = 0.71073 Å
*a* = 5.6867 (3) Å	Cell parameters from 1705 reflections
*b* = 12.2465 (8) Å	θ = 2.9–27.5°
*c* = 12.0831 (6) Å	µ = 0.13 mm^−^^1^
β = 113.150 (4)°	*T* = 100 K
*V* = 773.73 (8) Å^3^	Block, colourless
*Z* = 4	0.12 × 0.08 × 0.06 mm

### Morpholinium hydrogen oxalate (3) Data collection

**Table:**

Nonius Kappa CCD diffractometer	1769 independent reflections
Radiation source: Nonius FR591 rotating anode, Rotating Anode	1390 reflections with *I* > 2σ(*I*)
Graphite monochromator	*R*_int_ = 0.075
Detector resolution: 9.091 pixels mm^-1^	θ_max_ = 27.6°, θ_min_ = 3.3°
φ and ω scans to fill Ewald Sphere	*h* = −5→7
Absorption correction: multi-scan (SORTAV; Blessing, 1997)	*k* = −15→15
*T*_min_ = 0.887, *T*_max_ = 1.175	*l* = −15→15
6288 measured reflections	

### Morpholinium hydrogen oxalate (3) Refinement

**Table:**

Refinement on *F*^2^	Hydrogen site location: inferred from neighbouring sites
Least-squares matrix: full	H-atom parameters constrained
*R*[*F*^2^ > 2σ(*F*^2^)] = 0.042	*w* = 1/[σ^2^(*F*_o_^2^) + (0.0476*P*)^2^ + 0.2356*P*] where *P* = (*F*_o_^2^ + 2*F*_c_^2^)/3
*wR*(*F*^2^) = 0.110	(Δ/σ)_max_ < 0.001
*S* = 1.05	Δρ_max_ = 0.29 e Å^−^^3^
1769 reflections	Δρ_min_ = −0.26 e Å^−^^3^
111 parameters	Extinction correction: SHELXL2014 (Sheldrick, 2015bb), Fc^*^=kFc[1+0.001xFc^2^λ^3^/sin(2θ)]^-1/4^
0 restraints	Extinction coefficient: 0.128 (10)
Primary atom site location: structure-invariant direct methods	

### Morpholinium hydrogen oxalate (3) Special details

**Table:**

Geometry. All esds (except the esd in the dihedral angle between two l.s. planes) are estimated using the full covariance matrix. The cell esds are taken into account individually in the estimation of esds in distances, angles and torsion angles; correlations between esds in cell parameters are only used when they are defined by crystal symmetry. An approximate (isotropic) treatment of cell esds is used for estimating esds involving l.s. planes.

### Morpholinium hydrogen oxalate (3) Fractional atomic coordinates and isotropic or equivalent isotropic displacement parameters (Å^2^)

**Table:**

	*x*	*y*	*z*	*U*_iso_*/*U*_eq_	
O26	0.15522 (19)	0.29264 (8)	0.49858 (9)	0.0209 (3)	
O23	0.6961 (2)	0.22022 (8)	0.44496 (10)	0.0241 (3)	
H23	0.8454	0.2452	0.4678	0.036*	
O14	0.5274 (2)	0.36802 (9)	0.17836 (10)	0.0269 (3)	
O24	0.6384 (2)	0.36286 (9)	0.54702 (11)	0.0303 (3)	
O25	0.1977 (2)	0.17448 (9)	0.36656 (10)	0.0269 (3)	
N11	0.6060 (2)	0.57855 (10)	0.28681 (11)	0.0201 (3)	
H11A	0.6178	0.6219	0.3499	0.024*	
H11B	0.6287	0.6212	0.2301	0.024*	
C22	0.2790 (3)	0.24484 (12)	0.44633 (13)	0.0181 (3)	
C21	0.5606 (3)	0.28215 (12)	0.48638 (13)	0.0187 (3)	
C13	0.7757 (3)	0.41615 (13)	0.22554 (14)	0.0242 (4)	
H13A	0.8032	0.4566	0.1607	0.029*	
H13B	0.9063	0.3578	0.2541	0.029*	
C12	0.8090 (3)	0.49324 (12)	0.32829 (14)	0.0216 (4)	
H12A	0.7971	0.4522	0.3965	0.026*	
H12B	0.9797	0.5281	0.3563	0.026*	
C16	0.3484 (3)	0.52708 (13)	0.23515 (14)	0.0229 (4)	
H16A	0.2153	0.5841	0.2035	0.027*	
H16B	0.3167	0.4859	0.2984	0.027*	
C15	0.3368 (3)	0.45091 (13)	0.13495 (15)	0.0257 (4)	
H15A	0.1655	0.4166	0.0997	0.031*	
H15B	0.3629	0.4931	0.0708	0.031*	

### Morpholinium hydrogen oxalate (3) Atomic displacement parameters (Å^2^)

**Table:**

	*U*^11^	*U*^22^	*U*^33^	*U*^12^	*U*^13^	*U*^23^
O26	0.0142 (6)	0.0220 (5)	0.0276 (6)	0.0011 (4)	0.0095 (4)	−0.0038 (4)
O23	0.0133 (6)	0.0254 (6)	0.0359 (6)	−0.0027 (4)	0.0121 (5)	−0.0082 (5)
O14	0.0195 (6)	0.0230 (6)	0.0355 (7)	−0.0009 (5)	0.0078 (5)	−0.0072 (5)
O24	0.0223 (6)	0.0305 (6)	0.0433 (7)	−0.0095 (5)	0.0185 (5)	−0.0158 (5)
O25	0.0181 (6)	0.0322 (6)	0.0313 (6)	−0.0061 (5)	0.0107 (5)	−0.0122 (5)
N11	0.0204 (7)	0.0183 (6)	0.0230 (7)	−0.0013 (5)	0.0101 (5)	−0.0016 (5)
C22	0.0144 (8)	0.0174 (7)	0.0224 (8)	0.0008 (6)	0.0073 (6)	0.0010 (5)
C21	0.0157 (8)	0.0191 (7)	0.0226 (7)	−0.0010 (6)	0.0090 (6)	0.0015 (6)
C13	0.0152 (8)	0.0261 (8)	0.0302 (9)	0.0022 (6)	0.0075 (6)	−0.0026 (7)
C12	0.0156 (8)	0.0251 (8)	0.0240 (8)	0.0001 (6)	0.0077 (6)	0.0002 (6)
C16	0.0162 (8)	0.0256 (8)	0.0280 (8)	0.0008 (6)	0.0099 (7)	−0.0007 (6)
C15	0.0164 (8)	0.0299 (9)	0.0284 (9)	0.0011 (6)	0.0062 (7)	−0.0050 (6)

### Morpholinium hydrogen oxalate (3) Geometric parameters (Å, º)

**Table:**

O26—C22	1.2596 (18)	C22—C21	1.548 (2)
O23—H23	0.8400	C13—H13A	0.9900
O23—C21	1.3127 (18)	C13—H13B	0.9900
O14—C13	1.4263 (19)	C13—C12	1.511 (2)
O14—C15	1.4260 (19)	C12—H12A	0.9900
O24—C21	1.2062 (18)	C12—H12B	0.9900
O25—C22	1.2389 (18)	C16—H16A	0.9900
N11—H11A	0.9100	C16—H16B	0.9900
N11—H11B	0.9100	C16—C15	1.509 (2)
N11—C12	1.4902 (19)	C15—H15A	0.9900
N11—C16	1.4876 (19)	C15—H15B	0.9900
			
C21—O23—H23	109.5	C12—C13—H13B	109.3
C15—O14—C13	110.06 (11)	N11—C12—C13	109.36 (12)
H11A—N11—H11B	108.1	N11—C12—H12A	109.8
C12—N11—H11A	109.6	N11—C12—H12B	109.8
C12—N11—H11B	109.6	C13—C12—H12A	109.8
C16—N11—H11A	109.6	C13—C12—H12B	109.8
C16—N11—H11B	109.6	H12A—C12—H12B	108.3
C16—N11—C12	110.42 (12)	N11—C16—H16A	109.9
O26—C22—C21	114.79 (13)	N11—C16—H16B	109.9
O25—C22—O26	127.01 (14)	N11—C16—C15	108.91 (12)
O25—C22—C21	118.19 (13)	H16A—C16—H16B	108.3
O23—C21—C22	113.60 (12)	C15—C16—H16A	109.9
O24—C21—O23	125.08 (14)	C15—C16—H16B	109.9
O24—C21—C22	121.29 (13)	O14—C15—C16	110.95 (12)
O14—C13—H13A	109.3	O14—C15—H15A	109.4
O14—C13—H13B	109.3	O14—C15—H15B	109.4
O14—C13—C12	111.78 (12)	C16—C15—H15A	109.4
H13A—C13—H13B	107.9	C16—C15—H15B	109.4
C12—C13—H13A	109.3	H15A—C15—H15B	108.0

### Morpholinium hydrogen oxalate (3) Hydrogen-bond geometry (Å, º)

**Table:**

*D*—H···*A*	*D*—H	H···*A*	*D*···*A*	*D*—H···*A*
O23—H23···O26^i^	0.84	1.75	2.587 (2)	173
N11—H11*A*···O26^ii^	0.91	2.06	2.879 (2)	149
N11—H11*A*···O24^ii^	0.91	2.27	2.945 (2)	131
N11—H11*B*···O23^iii^	0.91	2.51	3.166 (2)	130
N11—H11*B*···O25^iii^	0.91	1.92	2.773 (2)	156
C12—H12*A*···O24	0.99	2.57	3.534 (2)	164
C12—H12*B*···O24^iv^	0.99	2.42	3.395 (2)	167
C16—H16*A*···O23^iii^	0.99	2.64	3.156 (2)	113
C16—H16*A*···O25^v^	0.99	2.43	3.378 (2)	161

Symmetry codes: (i) *x*+1, *y*, *z*; (ii) −*x*+1, −*y*+1, −*z*+1; (iii) −*x*+1, *y*+1/2, −*z*+1/2; (iv) −*x*+2, −*y*+1, −*z*+1; (v) −*x*, *y*+1/2, −*z*+1/2.
